# 

**Published:** 1888-07

**Authors:** 


### CONTENTS.

### COMMUNICATIONS.

Address ........ 317
Oral Antisepsis ........ 327
Practical Application of Electrolysis ........ 333
Art in Dentistry ........ 336
First Permanent Molars ........ 340
Nerve Transplantation from Animal to Man ........ 344
Crown and Bridge-Work ........ 345
Diseases Outside the Mouth that Affect the Oral Structures ........ 349
CORRESPONDENCE ........ 355-361
Dental College of the University of Michigan ........ 361
Ohio College of Dental Surgery ........ 362
Missouri Dental College ........ 362
Indiana Dental College ........ 363
University of Tennessee ........ 363
St. Louis College of Physicians and Surgeons ........ 364
University of Iowa ........ 364
Minnesota Hospital College ........ 364
Chicago College of Dental Surgeons ........ 365
SALMAGUNDI ........ 358

### EDITORIAL.

Ohio State Dental Society ........ 365
American and Southern Dental Association ........ 365
Mad River Valley Dental Association ........ 366
Rhinological Association ........ 367
Hymenial ........ 367
The Six-Year-Old Molars ........ 367

